# Embryonic esophageal rhabdomyosarcoma in an adult male: A case report and literature review

**DOI:** 10.3389/fonc.2022.951433

**Published:** 2022-09-02

**Authors:** Xiaoman Zhang, Guang Li

**Affiliations:** Department of Radiation Oncology, The First Hospital of China Medical University, Shenyang, China

**Keywords:** esophageal rhabdomyosarcoma, esophagus, radiotherapy, chemotherapy, prognosis

## Abstract

**Basic principle:**

There exists a rare aggressive neoplasm called esophageal rhabdomyosarcoma. It originates in cells of the striated muscle or mesenchymal cells which differ from the striated muscle. This tumor has a high degree of malignancy and extensive metastasis. Masses of the sick people are at a high phase when going to hospital. Consequently, the prognosis is exceedingly bad.

**Patient concerns:**

A 54-year-old male presented with dysphagia as the initial symptom. Gastroscopy showed an irregular protuberant lesion about 18–22 cm from the incisor. The lesion was observed to be pathological under gastroscopy and was diagnosed as an (esophageal) sarcoma.

**Diagnosis:**

Embryonic esophageal rhabdomyosarcoma.

**Interventions:**

After receiving two cycles of DP (docetaxel and cisplatin) chemotherapy in the local hospital, the patient received 60 Gy of radiotherapy in 30 fractions combined with chemotherapy at our hospital. Dysphagia was relieved, and the tumor appeared significantly shrunken on imaging after the treatment.

**Outcomes:**

Lung metastasis occurred 1 month after radiotherapy, and the patient died of pulmonary edema on March 11, 2022.

**Lessons:**

Previously reported cases of embryonic esophageal rhabdomyosarcoma are few. Theoretically, the disease should occur in adolescents; nevertheless, our case was a man who was in middle-aged; the neoplasm was in an unusual position: the upper part of the esophagus. Moreover, the patient initially had good response to the combination of radiotherapy and chemotherapy. Although he died 8 months after diagnosis, the presented data represent a valuable resource for understanding the survival benefits of treating embryonic esophageal rhabdomyosarcoma patients with radiotherapy combined with chemotherapy. In addition, we reviewed the previously reported literature, and a total of 17 cases of esophageal rhabdomyosarcoma were identified and analyzed.

## Introduction

Adenocarcinoma and squamous cell carcinoma account for the majority of esophageal malignancies. Esophageal sarcoma is rare, accounting for only about 0.5% of esophageal cancers (1–6). Among esophageal sarcomas, fibrosarcoma is the most common pure sarcoma, while leiomyosarcoma and rhabdomyosarcoma are extremely rare (7). Esophageal rhabdomyosarcoma (RMS) is a rare tumor. Wolfensberger et al. first reported a case of primary esophageal rhabdomyosarcoma in 1894 and identified rhabdomyosarcoma under an electron microscope (8). The World Health Organization (WHO) categorizes rhabdomyosarcoma into pleomorphic, acinar, and embryonic types. Pleomorphic rhabdomyosarcoma is the most common type, which tends to occur in the elderly and has the best prognosis. Acinar type is more common in young people and is highly malignant but sensitive to radiotherapy. Embryonic type is the most rare and mainly occurs in infants or young people (9); its prognosis is between that of the pleomorphic and acinar types. The clinical symptoms of esophageal rhabdomyosarcoma are similar to those of esophageal cancer: progressive dysphagia, retrosternal pain, nausea and vomiting, and weight loss. By the virtue of the shortage of feature clinical representations, the preoperative diacrisis ratio of the present inspection approaches is excessively low. Besides, RMS is frequently misdiagnosed to be the esophageal cancer. Furthermore, diagnosis of this disease under a light microscope is extremely difficult (10). The treatment of this disease is mainly radical surgery; esophagectomy or esophagogastric resection is the first choice (2). Lymph node dissection should be performed routinely during the operation to reduce local recurrence and distant metastasis of the tumor (11). Radiotherapy or chemotherapy should be administered according to the specific situation after the operation. At present, most of the research on esophageal rhabdomyosarcoma comprises case reports. It is reported that a case of a middle-aged man suffering from the embryonic esophageal rhabdomyosarcoma can give a correct prognostic foundation for clinical diacrisis as well as cure.

## Case presentation

**Figure 1:**
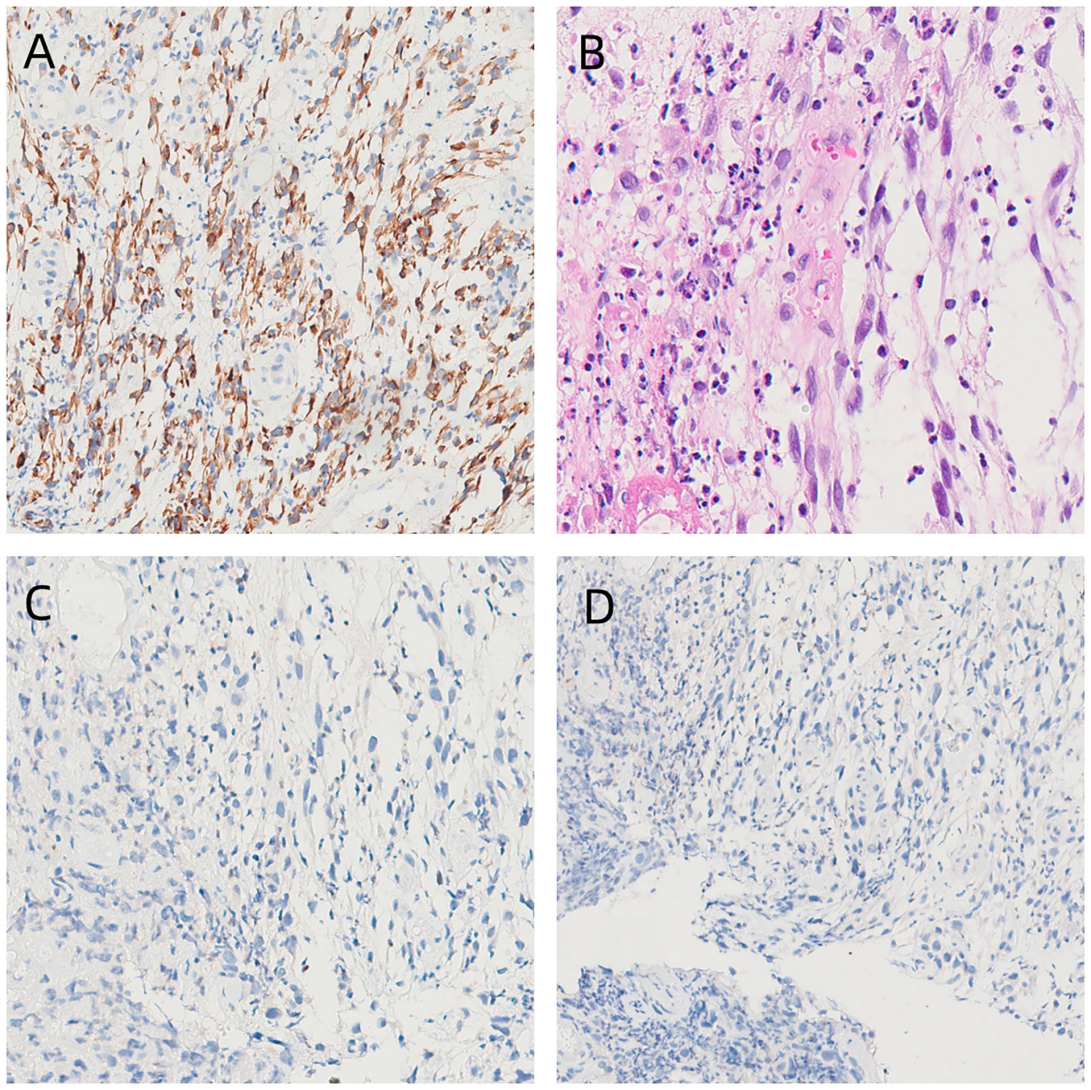
Histological and immunohistochemical findings. **(A)** Immunohistochemical staining for desmin shows positivity (×100), which indicates sarcoma. **(B)** HE staining (×100) of embryonic rhabdomyosarcoma. **(C)** Immunohistochemical staining for MyoD1 shows negativity (×100). **(D)** Immunohistochemical staining for myogenin shows negativity (×100).

The patient was a 54-year-old retired male of Han nationality. He was 170 cm tall and weighed 55 kg. His KPS (the Karnofsky Performance Status, for short) score reached 80. The sick man experienced a 20-cigarette-per-day smoking history lasting for 5 years and a drinking history of 8 bottles every day lasting for 30 years. He possessed a history of squamous cell tumor of the tongue and experienced one surgery in 2012. In addition, his father died from the lung cancer. The sick man was in hospital in Yingkou Central Hospital because of the dysphagia in August 2021. The gastroscopy illuminated an abnormal protuberant damage roughly from 18cm to 22 cm from the incisor. Besides, the surface was covered by a layer of the white moss. Pathological examination under gastroscopy showed an (esophageal) malignant tumor. The result of pathological consultation in our hospital was (esophageal) sarcoma. Immunohistochemistry showed vimentin (+), CK (-), CD117 (a few scattered +), CD34 (vascular +), SMA (-), DOG-1 (±), desmin (+), Ki-67 (about 10% +), S-100 (-), CD68 (histiocyte +), ERG (-), INI-1 (+), CD3 (T cell +), H-caldesmon (-), CD20 (-), CD31 (+), CD30 (-), P63 (-), myogenin (-) and MyoD1 (-). The immunohistochemical results suggested embryonic rhabdomyosarcoma (**Figures 1A–D**). Improved CT of the chest illustrated the ground-glass nodules and esophageal space-occupying damages within the center lobe of the right lung. A CT of the neck illuminated the postoperative variances within the right neck. Besides, we can see the lymph nodes of the left neck. The TNM stage before treatment was T3N0M0 (according to the latest eighth edition of AJCC staging system). The patient started chemotherapy at Yingkou Central Hospital on September 28, 2021. For economic reasons, the patient and his family refused to apply albumin paclitaxel and requested the application of the Medicare-reimbursed drug docetaxel. On October 1, 2021, he finished two cycles of DP (docetaxel and cisplatin) chemotherapy. During chemotherapy, the patient did not have any serious adverse reactions, and the effect was evaluated as SD(Stable Disease) after the last chemotherapy treatment.

On October 28, 2021, the patient arrived at our hospital for reexamination *via* cervical lymph node ultrasound. Several echogenic lymph nodes were seen in the right neck; the largest lymph node was about 0.67 × 0.47 cm and was located in Area II. Several lymph nodes echoes were seen in the left neck; the largest one was about 1.24 × 0.31 cm and was located in Area II. The lymph nodes were strip-shaped, and portal-like structures were seen. No obviously enlarged lymph nodes were found in the superior and inferior fossae of both clavicles. Ultrasound examination showed echoes of lymph nodes in Area II of the neck bilaterally (grade 2), and echoes of lymph nodes in Area IV of the left neck (grade 3). On November 1, 2021, esophageal spot film (DR) (barium) examination revealed a 4–5-cm-long lesion on the upper part of the esophagus, which suggested a high possibility of a space-occupying lesion in the upper esophagus.

**Figure 2:**
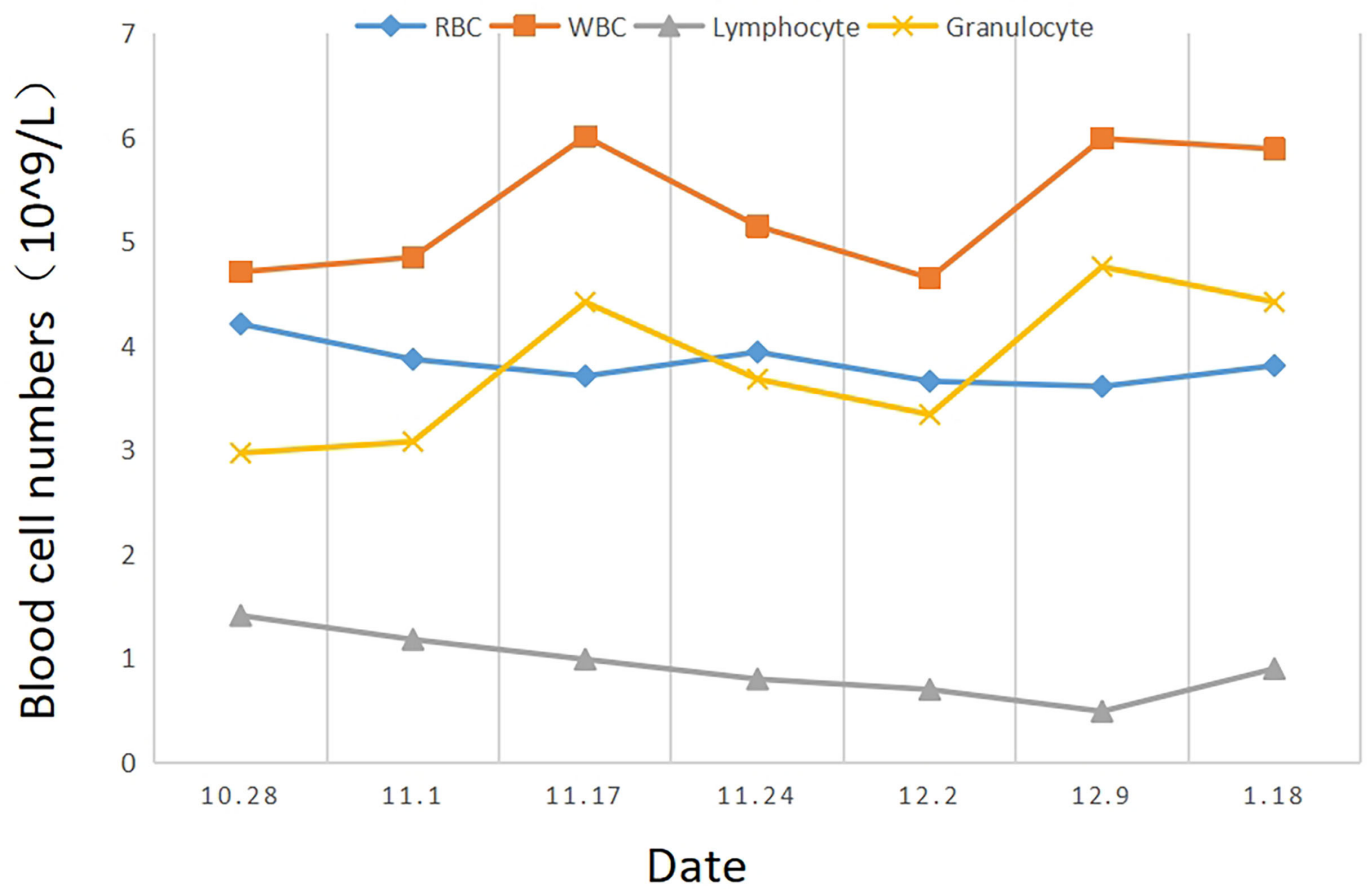
The changes in complete blood count (RBC, WBC, lymphocyte, and granulocyte) depended on the date (October 28, 2021-January 18, 2022).

**Figure 3:**
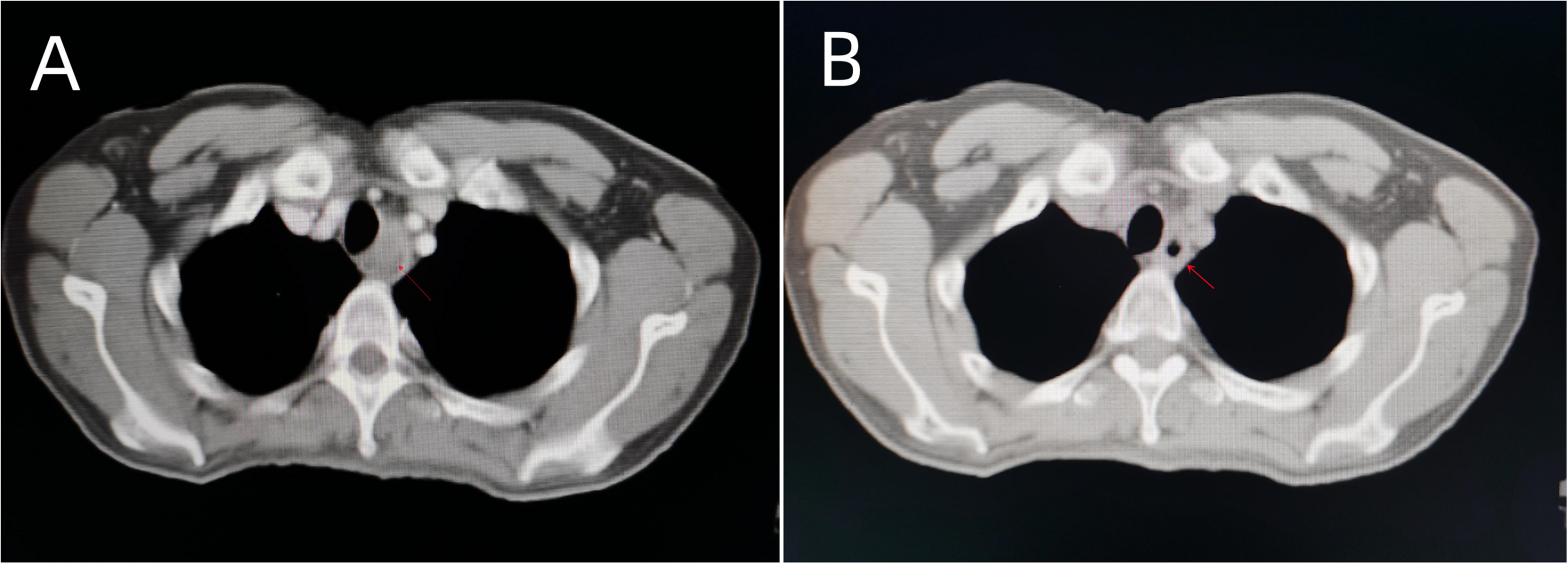
Chest CT before **(A)** and after **(B)** 60Gy/30 fractions of radiotherapy combined with chemotherapy, the tumor shrank significantly on the radiograph.

**Figure 4:**
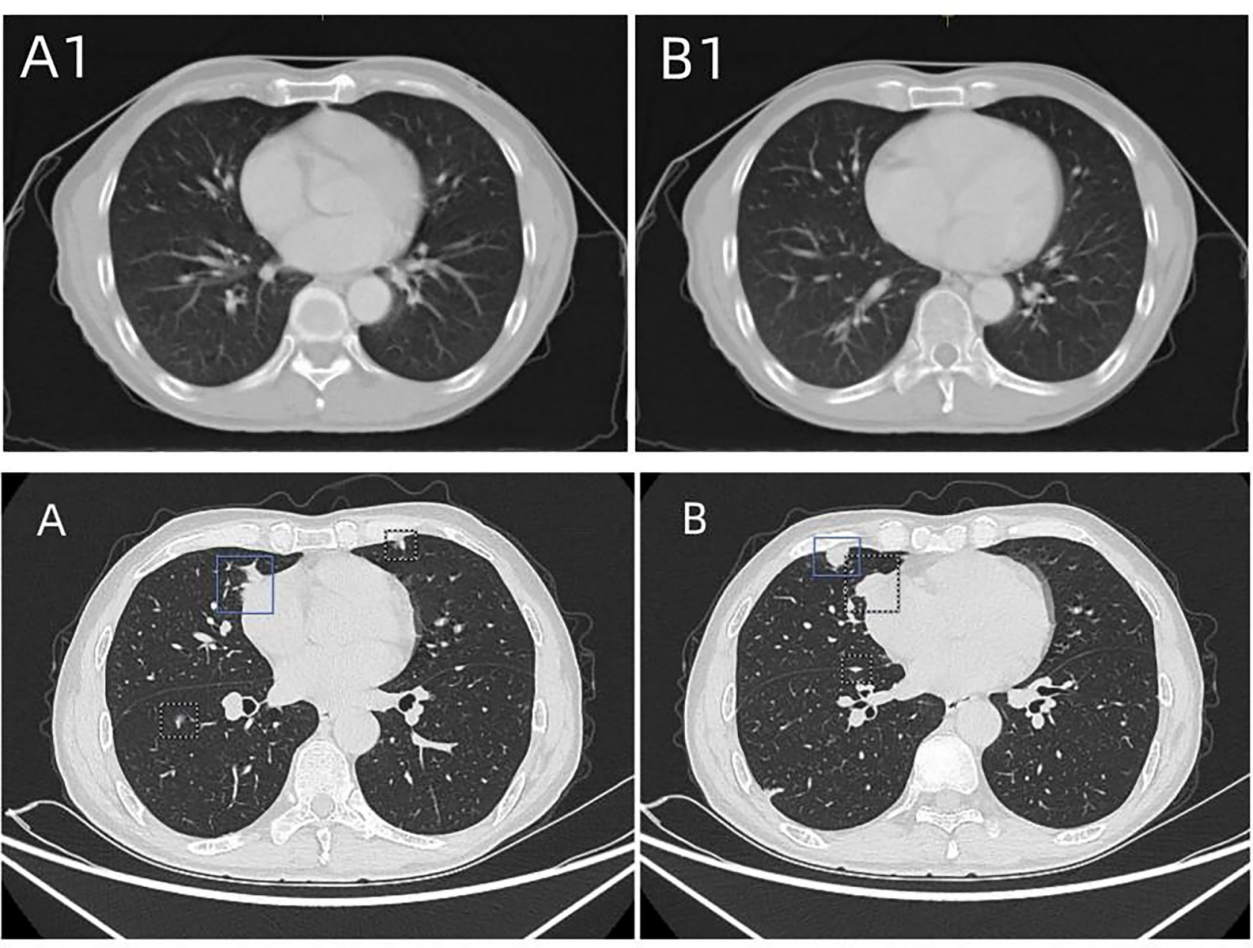
**(A1, B1)** Chest CT taken on October 29, 2021, before concurrent radiotherapy and chemotherapy. No obvious lung metastasis was found. **(A, B)** Chest CT taken on January 18, 2022, showing scattered nodules of different sizes in both lungs. The largest nodule was located near the heart border in the middle lobe of the right lung, which indicated pulmonary metastasis.

Intensity-modulated radiotherapy combined with chemotherapy was administered on November 3, 2021. Specifically, teggio capsule 40 mg bid was taken orally. During treatment, the patient had pain at the irradiation site, which was tolerable, and no drug treatment was given. The symptoms were relieved 2 weeks after the cessation of radiotherapy. The changes in whole blood-cell count during radiotherapy are shown in **Figure 2**. During the treatment, the patient’s red blood cell, white blood cell, and granulocyte counts were normal, and the lymphocyte count decreased only slightly. Overall, the hemogram of the patient was not greatly affected by radiotherapy and chemotherapy. After 60 Gy of radiotherapy administered in 30 fractions, the tumor was significantly shrunken on CT scan (**Figure 3**), and the patient’s symptoms of dysphagia were alleviated. The final dose of radiotherapy was administered on December 14, 2021, and regular reexaminations were conducted after the cessation of treatment. The patient’s reexamination on January 18, 2022, indicated pulmonary metastases (**Figures 4A, B**). Enhanced CT of the lung showed that scattered nodules of different sizes in both lungs. The largest nodule was located near the heart border of the middle lobe of the right lung; it was about 1.7 × 0.9 cm in size, had a CT value of 25 HU, and the CT value of the enhanced scan was about 27–70 HU. Cord shadow cand small nodule shadows could be seen in both lungs, and slightly enlarged lymph nodes were seen in the left and right hila. The wall of the upper esophagus was thickened with enhancement, and the surface was irregular. The diagnosis based on the imagining was multiple metastases in both lungs. Ultrasound examination showed echoes of lymph nodes in Area II of the left neck (grade 2) and Area IV of the left neck (grade 2–3). On January 21,2022, esophageal spot film reexamination showed that a space-occupying lesion persisted in the upper esophagus; however, the volume of the mass was reduced, and esophageal stenosis was slightly alleviated compared with the original film. After receiving supportive symptomatic treatment at the local hospital, dyspnea symptoms occurred and gradually aggravated in March. A plain chest scan performed in the local hospital revealed bilateral, arc-shaped liquid shadow in the thorax that were dominant in the right lung, which suggested bilateral pleural effusion. At the local hospital, the sick man was cured by pleural effusion drainage some times, with an entire drainage of roughly 9000 cc. The patient succumbed due to disease progression on March 11, 2022, and the overall survival time was 8 months.

## Review and discussion

Esophageal rhabdomyosarcoma is a rare malignant tumor of the esophagus. To date, only around 10 cases have been reported in the literature. In humans, only the upper one-third of the esophagus is composed of striated muscle, the central part of the esophagus contains both striated muscle and smooth muscle tissue, and the lower one-third of the esophagus is composed exclusively of smooth muscle. Esophageal rhabdomyosarcoma most often occurs in the middle to distal end of the esophagus (12). Nevertheless, a large quantity of researchers suppose that the neoplasm might stem from uniform mesenchymal cells which are placed in the foregut in the course of the embryonic evolution (12, 13). The median age at diagnosis of esophageal rhabdomyosarcoma is 58 years (26–76 years) (14). Among the reported cases, the age ranged from 41 to 82 years, with most patients being male. Pleomorphic rhabdomyosarcoma represents most of these reported cases; embryonic esophageal rhabdomyosarcoma is the least reported. To date, only two elderly male patients have been reported. Our case is a 54-year-old male and is the youngest of the embryonic cases reported thus far. Moreover, among the reported cases, the lesions were located in the upper segment of the esophagus in only three. Our case seemed to be this rare category. Also, it was the only case of embryonic esophageal rhabdomyosarcoma, and the damages placed in the upper esophagus part. The clinical symptoms of esophageal rhabdomyosarcoma are similar to those of esophageal cancer. The most common symptoms are progressive dysphagia (15), retrosternal pain, nausea and vomiting (16), and weight loss (15). However, compared with esophageal cancer, rhabdomyosarcoma is associated with less severe eating-obstruction symptoms, slower obstruction progress, and the obstruction symptoms are out of proportion to the size of the tumor (17). Barium examination of esophageal rhabdomyosarcoma may show a large intramural mass with surface ulceration (18), and some cases may rarely show signs of esophageal stenosis. CT or MRI can show uneven enhancement of the intramural mass (14). Rhabdomyosarcoma is usually composed of undifferentiated cells, and it is thus difficult to diagnose under a light microscope (10). Among the cases reported in recent years, only a few cases showed obvious cross-striation under a light microscope (7, 8, 10, 12, 19–21). In contrast, diagnosis is relatively easy using an electron microscope. The diagnosis can be made by the presence of actin (60–80 A) and myosin (120–150 A) filaments, notably the hexagonal arrangement of these filaments on cross section (10). Due to the rarity of esophageal rhabdomyosarcoma, there is currently no effective treatment. Surgery is the first choice of treatment (14). The survival time of patients who are diagnosed and treated early is relatively long. Almost all the reported cases were treated with surgery; four cases were treated with concurrent radiotherapy and chemotherapy. Esophagectomy or esophagogastric resection is the first choice (22), followed by endoscopic resection. Palliative surgery to reduce dysphagia, such as stent implantation, can improve quality of life (23). The factors affecting survival rate include the integrity of the surgical resection, growth mode, postoperative stage, tumor grade, and tumor location (15). Whether the tumor can be completely removed *via* surgery has a significant impact on prognosis.

PubMed databases were searched for literature of esophageal rhabdomyosarcoma, The following keywords were used: (((((“Esophagus”[Mesh]) OR (esophagus[Title/Abstract])) OR (gullet[Title/Abstract])) OR (oesophagus[Title/Abstract])) AND (((((“Rhabdomyosarcoma”[Mesh]) OR (Rhabdomyosarcoma[Title/Abstract])) OR (rhabdosarcoma[Title/Abstract])) OR (rhabdomyoma sarcomatosum[Title/Abstract])) OR (Rhabdomyosarcomas[Title/Abstract]))) OR ((Esophageal rhabdomyosarcoma[Title/Abstract]) OR (rhabdomyosarcoma of the esophagus[Title/Abstract])), and 63 results were retrieved. After removing duplicates and irrelevant studies, finally, 17 cases were identified. In addition, cases with unclear pathological classification or lack of follow-up information were excluded. According to the reported cases, the clinical characteristics, treatment, and prognosis of patients with esophageal rhabdomyosarcoma are shown in **Table 1** (1–3, 7, 9, 10, 12, 17, 19, 24–30).Due to the limited number of cases of this disease and because only a small number of selected cases survived, reliable statistical results may not be available to guide treatment. Nevertheless, it could be known according to the data that esophageal rhabdomyosarcoma owns a poor prognosis as well as a highly mortality ratio. In researched cases, most of sick people died of the distant metastases or recurrence after the cure, except for some cases without follow-up records. In comparison, patients with operable lower esophageal sarcoma had a better prognosis than patients with inoperable upper esophageal sarcoma. Therefore, the reasons for the poor prognosis of the disease may be as follows: 1. the anatomical structures surrounding the esophagus lead to the reduced complete-resection rate of the tumor and the limitations of surgical treatment; 2. soft tissue sarcoma has a high degree of malignancy and low sensitivity to radiotherapy and chemotherapy; and 3. there exist few researches in relation to the pathomechanism of esophageal rhabdomyosarcoma, the particular molecular system is still unknown, and efficient targeted therapy along with the immunotherapy is unusable.

**Table 1:** Clinical features, treatments, and prognosis of the esophageal rhabdomyosarcoma.

Sex	Age	Type	Location	Size (cm)	Treatment		Metastasis	Prognosis	Publication year
F	55	pleomorphic rhabdomyosarcoma	in the lower half of the esophagus.	10×4.5×4.5	Surgery, radiotherapy, chemotherapy		yes	8 months (dead)	2006
M	55	pleomorphic rhabdomyosarcoma	at 25 cm	/	radiotherapy, chemotherapy		yes	6 months (dead)	1991
M	63	pleomorphic rhabdomyosarcoma	at 36 cm	/	Surgery, radiotherapy, chemotherapy		no	20 months (dead)	2011
M	61	pleomorphic rhabdomyosarcoma	lower third of the oesophagus	12×4.0	Surgery		no	1 months (dead)	1980
M	61	embryonic rhabdomyosarcoma	25–29 cm in the mid-oesophagus	8.5×3.5×2.5	Surgery, radiotherapy, chemotherapy		yes	9 months (alive)	2012
M	46	pleomorphic rhabdomyosarcoma	at 25 cm	13×8.0	Surgery		no	3 years (alive)	2001
M	65	botrytis rhabdomyosarcoma	the middle esophagus	4.0×2.0×1.5	Surgery		/	/	1992
M	82	embryonic rhabdomyosarcoma	the middle esophagus	9.0	/		yes	dead	1986
M	50	pleomorphic rhabdomyosarcoma	the lower esophagus	8.0×4.0×6.0	Surgery		yes	1 months (dead)	1998
M	46	pleomorphic rhabdomyosarcoma	the middle and lower esophagus	10×3.0×1.5	Surgery		/	/	2002
M	44	/	the middle and lower esophagus	10×3.0	Surgery		/	/	1977
F	51	acinar rhabdomyosarcoma	the middle esophagus	4.0×3.0×2.0	Surgery		/	/	1998
M	49	acinar rhabdomyosarcoma	at 23 cm	4.0×3.5×1.5	Surgery		yes	5 months (dead)	1996
M	41	pleomorphic rhabdomyosarcoma	cervical esophagus	5.0×3.0×3.0	Surgery, chemotherapy		yes	6 months (alive)	2002
M	65	pleomorphic rhabdomyosarcoma	the middle esophagus	8.0×3.0×3.0	Surgery		/	/	1995
M	48	/	the middle esophagus	3.5×4.0×3.0	Surgery		yes	8 months (dead)	1994
M	61	pleomorphic rhabdomyosarcoma	deep surface of right lobe of thyroid	5.0×4.0×4.0	Surgery		/	/	2014
M	65	pleomorphic rhabdomyosarcoma	the middle esophagus	8.0×3.0×3.0	Surgery	/		/	1995
M	48	/	the middle esophagus	3.5×4.0×3.0	Surgery	yes		8 months (dead)	1994
M	61	pleomorphic rhabdomyosarcoma	deep surface of right lobe of thyroid	5.0×4.0×4.0	Surgery	/		/	2014

Recently, etoposide (Vp-16), Adriamycin (ADM), DPP, dacarbazine (DTIC), ifosfamide (IFO), and taxus have obtained satisfactory consequences during the chemotherapy for soft tissue sarcomas. Recent studies have shown that the combined chemotherapy of anthracycline drugs (e.g., ADM and EPI) and IFO seems to be the most effective for the treatment of advanced soft tissue sarcoma (28). He Shengxiu et al. reported a case of pleomorphic esophageal rhabdomyosarcoma with tumor recurrence and metastasis after operation. After two courses of DDP + VP-16 + ADM chemotherapy, the curative effect was not obvious. Two courses of EPI + IFO chemotherapy was then administered, and the condition improved (28). S. Patricia et al. reported a case of pleomorphic esophageal rhabdomyosarcoma in which the patient’s condition was stable after receiving four cycles of ADM + IFO chemotherapy (2). Though the sick people died unpredictably from the stent blockage, he had lived for twenty months from the diagnosis date had a great quality of life, with no dysphagia. In general, for patients with esophageal rhabdomyosarcoma, the combination of ADM or EPI with IFO and surgery or radiotherapy combined with this plan in inoperable cases should be attempted to prolong survival.

Regarding the histological origins of esophageal rhabdomyosarcoma, some scholars have suggested the following mechanisms: 1) myogenic metaplasia of primitive mesenchymal cells; 2) metaplasia of other connective tissue cells; or 3) ectopic migration of rhabdomyoblasts in the pharyngeal and upper esophageal regions (24). Nevertheless, there exists a shortage of studies about the disease molecular system. So, no targeted therapy or mature immune has been disclosed. In years to come, we might be able to deeply research and catch on the pathomechanism of esophageal rhabdomyosarcoma for the purpose of making profound breakouts when treating this sickness, giving hopes to extend the lifetimes for more sick people.

Though cases of esophageal rhabdomyosarcoma have been recorded in the literature, embryonic cases placed in the upper esophagus segment are still seldomly disclosed during the last five years. In other cases, most of the tumors were located in the middle and lower esophagus and were treated surgically, and the role of radiotherapy in its treatment was not clearly pointed out. Our case disclosure owns a distinct radiotherapy process. Besides, the notable therapeutic impact attained by the radiotherapy has an obvious picture back-up. This is of great significance for our future treatment of inoperable embryonic rhabdomyosarcoma of the upper esophagus. In the future, we can also study more the effect of radiotherapy time, radiotherapy dose, and the combination of radiotherapy and chemotherapy on embryonic esophageal rhabdomyosarcoma in the upper esophagus, and explore more effective treatment schemes to provide the possibility of survival for such patients who have lost the opportunity of surgery.

However, our report also has some limitations: 1. Some treatment details of patients outside our hospital cannot be reported in detail due to the lack of data. 2. At the follow-up visit, the patient had died and could not be recorded from the perspective of the patient himself, which could only be replaced by his family members. 3. When we evaluated the curative effect, we only did lung CT at the end of radiotherapy for comparison, but did not conduct imaging evaluation every week during radiotherapy. 4. For the previous treatment, there may be some bias due to the memory bias of the family members.

## Patient perspective

My husband suffered from two circles of chemotherapy after diacrisis in the local hospital, however, his dysphagia didn`t shorten obviously. Consequently, we came with the aim of carrying on his cure. Doctors drew up a thorough schedule of radiotherapy for him. Simultaneously, my husband’s dysphagia alleviates notably behind the treatment. Though he unavoidably underwent the lung metastasis, he died after the treatment in the local hospital in March this year, we are excessively thankful to doctors for their helping to reduce my husband`s pain. I agreed to share my medical history and signed an informed consent form.

## Conclusion

In summary, we report a case of embryonic esophageal rhabdomyosarcoma in an adult male whose lesions were located in the upper segment of the esophagus. Although he died of lung metastasis after treatment, the combination of radiotherapy and chemotherapy had achieved favorable results and this case is expected to provide therapeutic reference for future patients. We reviewed the origin, epidemiological characteristics, clinical manifestations, diagnosis, and treatment of esophageal rhabdomyosarcoma. In addition, we summarized the reported cases of esophageal rhabdomyosarcoma, determined that the disease has a high degree of malignancy and a very low average survival rate and summarized several possible reasons for these features. If operation can be performed early and combined with comprehensive treatment, we can expect to prolong the survival time of patients (31). It was also observed that combination chemotherapy with anthracycline drugs (e.g., ADM, EPI) and IFO may play a role in effective treatment of esophageal rhabdomyosarcoma, and their application is worthy of more attempts. The important role of radiotherapy is affirmed during the embryonic esophageal rhabdomyosarcoma treatment in this case. The prospects for deep precise radiotherapy is brought forward, which offers the powerful proofs for treating sick people with unpractical embryonic esophageal rhabdomyosarcoma of the upper part of the esophagus. The molecular mechanism of esophageal rhabdomyosarcoma and its potential therapeutic targets require more research. Although the sample amount of cases is constrained, this study is going to offer worthy data for the management and treatment of analogous sick people with esophageal rhabdomyosarcoma in the upper part of the esophagus.

## Data availability statement

The original contributions presented in the study are included in the article/supplementary material. Further inquiries can be directed to the corresponding author.

## Ethics statement

Written informed consent was obtained from the individual(s) for the publication of any potentially identifiable images or data included in this article.

## Author contributions

XZ and GL independently completed the follow-up, information collection, literature review, manuscript drafting and proofreading together.

## Acknowledgments

We would like to thank the researchers and study participants for their contributions. The authors did not receive funding for this research.

## Conflict of interest

The authors declare that the research was conducted in the absence of any commercial or financial relationships that could be construed as a potential conflict of interest.

## Publisher’s note

All claims expressed in this article are solely those of the authors and do not necessarily represent those of their affiliated organizations, or those of the publisher, the editors and the reviewers. Any product that may be evaluated in this article, or claim that may be made by its manufacturer, is not guaranteed or endorsed by the publisher.
